# Finnish university physics teachers’ experiences of transferring to online teaching due to COVID-19 pandemic

**DOI:** 10.1007/s43545-023-00647-1

**Published:** 2023-03-23

**Authors:** Risto Leinonen, Mikko H. P. Kesonen, Mervi A. Asikainen

**Affiliations:** 1 Department of Physics and Mathematics, University of Eastern Finland, Joensuu, Finland; 2 Faculty of Education and Culture, Tampere University, Tampere, Finland

**Keywords:** Physics teacher, University level, COVID-19, Experiences

## Abstract

The COVID-19 pandemic arguably caused the most rapid and extensive educational reform ever witnessed. This article addresses the challenges, possibilities, and consequences that the pandemic has set for Finnish physics university teachers. The data were gathered with a literature-based online survey consisting of Likert scale claims and open-ended questions related to the following themes: the use of technology, interaction, learning and evaluation, experimental working, and the future. In 2021, the survey was distributed to all Finnish universities where physics is taught, and 52 responses were received. The results show the biggest challenges were related to the different aspects of interaction, experimental working, and adjusting evaluation. The opportunities were less recognized but the versatility and flexibility of instruction and the use of technology in the evaluation became apparent in the teachers’ responses. The consequences of pandemic time for physics teaching and learning were seen rather negatively, even if a majority of teachers intended to change their old teaching practices in the future. The results indicate that university teachers need more discipline-based pedagogical support for enhancing learning, interaction, and well-being as the physics instruction likely gets more versatile after the pandemic.

**Supplementary Information:**

The online version contains supplementary material available at 10.1007/s43545-023-00647-1.

## Introduction

Since the COVID-19 pandemic started in March 2020, research communities have made great efforts whilst aiming to figure out and tackle the pandemic-related issues: this has not been limited to the epidemic and medical research, but the pandemic has influenced other scientific disciplines as well, including educational research.

O’Brien (2021) reports that teachers might lack the necessary skills for using the equipment required in online teaching. Ambivalently, the study of Lemay et al. (2020) revealed that a majority of the K-12 teachers from various disciplines (applied health science, arts and humanities, business and management, communication, media, and journalism, education, science, technology engineering and mathematics, and social science) lacked previous experience of online teaching but still felt secure in adopting online teaching technology. Teachers experienced teaching to be equally demanding remotely. Besides, teaching became a more important aspect of their duties than before pandemic. Teachers felt motivated, and they were able to follow their original values and objectives, even if the amount of time needed for teaching increased. However, less than a half of teachers stated willingness to teach solely online in the future (Lemay et al. 2020). Interestingly, the study of Trust and Whalen (2021) showed that teachers from different school levels have faced challenges with the following technology-related issues: finding, evaluating, and choosing appropriate digital tools, access to technology, how to teach online, general technology concerns (such as screen time fatigue), and student use and parental support of technology use. Then again, the study with university teachers from various disciplines showed that even if the examined teachers lacked the confidence for online instruction, later they recognized some possibilities it can offer (Tsegay et al. 2022).

Regarding the interaction in online teaching, a small majority of teachers found it easier to keep track of students’ progress and to resolve their challenges. Only a minority of the teachers reported having felt disconnected from their students in online teaching. (Lemay et al. 2020) This finding is supported by Kannan et al. (2020) who have reported a better engagement over internet whilst a specially designed intervention for remote teaching has been utilised. Then again, the sense of community and the number of discussions has declined in remote teaching (Dew et al. 2021; Romadhon et al. 2021; Trust and Whalen 2021). O’Brien (2021) points out that whilst interacting with students online, one should keep the resources and equality of students in mind.

Teachers have reported employing versatile and various teaching strategies and methods during the pandemic: e.g., getting students to answer questions, online quizzes and exams, projects done independently or in groups, live video classes, giving presentations, instructional videos, and students familiarising themselves with materials (Lemay et al. 2020; Trust and Whalen 2021). With respect to evaluation, Romadhon et al. (2021) have reported a reduction in using multiple ways of evaluation during pandemic. Rapanta et al. (2020) suggest that continuous evaluation should be emphasised instead of summative evaluation.

Teachers have highlighted some limitations concerning online instruction. They are related to overburdening the students, lack of technology or familiarity with technology, lack of knowledge of instructional design, and concern about students cheating (e.g., Lemay et al. 2020; Trust and Whalen 2021). Experimental working has been difficult to implement during the pandemic, but it has been substituted with online laboratories, simulations, and homemade experiments, also in the field of physics (Jelicic et al. 2022; Lemay et al. 2020; O’Brien 2021; Romadhon et al. 2021).

Teachers state that learning outcome did not lose its significance during the pandemic, the quality of students’ work did not decrease, there was not much more cheating amongst students, and pandemic time teachings did not have negative effects on students’ future studies. (Lemay et al. 2020). Kannan et al. (2020) have reported a better learning outcome in online teaching implemented with a specially designed intervention in comparison to conventional instruction. Then again, Hasan and Odja (2021) have reported that mixing synchronous online and face-to-face instruction has left students with poor problem-solving skills. Delgado (2021) has revealed that online courses have a bigger drop-off rate than face-to-face courses.

Even if research communities have revealed valuable findings related to pandemic time teaching and learning, there is still a need for research related to teachers’ experiences from individual disciplines as these types of studies can give insight about subject-specific challenges. This article addresses Finnish physics university teachers’ experiences concerning teaching in pandemic time, and the research questions are formulated as follows:

1. *What sorts of challenges did university teachers face in teaching physics during the pandemic?*
2. *What sorts of possibilities did university teachers experience in teaching physics during the pandemic?*
3. *What sorts of changes will remain in teaching physics at university due to the pandemic?*

## Methods

### Constructing the survey

The data were gathered with a self-constructed online survey. The process started when the authors familiarised themselves with the existing surveys (e.g., Motz et al. 2021) to evaluate their suitability for the focus of this research and to evaluate the themes found in them. Second, the content in the research articles related to issue at hand were analysed (Kohlbacher 2016), and the most prominent findings were categorised to find the emerging themes related to pandemic time instruction from the perspective of university level physics teachers. The main themes and their descriptive features are summarized in Table 1.

**Table 1:** The main themes with citations and their descriptive features related to pandemic time teaching from the perspective of university level physics teachers

Theme and citations	Descriptive features
Using technology (Ahmed and Gwamna 2020; Dew et al. 2021; Hasan and Odja 2021; Kannan et al. 2020; Lemay et al. 2020; O’Brien 2021)	Challenges and opportunities related to using technology in teaching Support needed and received
Interaction (Delgado 2021; Lemay et al. 2020; O’Brien 2021; Rapanta et al. 2020; Romadhon et al. 2021)	Challenges and opportunities related to interaction in online teaching Changes in the amount and modes of interaction Changes in giving feedback The sense of community
Learning and evaluation (Campari et al. 2021; Dew et al. 2021; Kannan et al. 2020; Klein et al. 2021; O’Brien 2021; Puspisatari et al. 2021; Rapanta et al. 2020; Romadhon et al. 2021)	Challenges and opportunities related to evaluation in online teaching Changes in learning Changes in evaluation
Experimental working (Campari et al. 2021; Klein et al. 2021; Lemay et al. 2020; O’Brien 2021; Romadhon et al. 2021)	Challenges and opportunities related to experimental working during pandemic Changes in experimental working
Other	Other challenges and opportunities related to pandemic time teachings emerging from teachers
Future	Future of teaching in post-pandemic world

The aforementioned themes served as a base for constructing the survey found in Online Appendix. The survey items were constructed so that each theme was addressed with Likert scale claims and open-ended questions. These items were then mirrored to the themes seen in Table 1 so that all the themes found from the literature are addressed. Besides these themes, teachers were asked background information concerning their universities, students, degree and title, and teaching methods used during the pandemic.

For quantitative part of the study, each theme was addressed with 3–6 Likert scale claims (1 = Totally disagree, 2 = Disagree, 3 = Neutral, 4 = Agree, 5 = Totally agree) so that all the descriptive features (see Table 1) were covered in the survey. Respondents had an opportunity to justify or specify their choices in open-ended question to enrich the data with qualitative elements (Creswell 2009). Other qualitative element, namely open-ended questions were used to ask teachers about the challenges and possibilities related to the themes. Regarding the theme Learning and evaluation, respondents were also asked about the actual implementation and changes of evaluation. Solely open-ended questions were presented in the theme Other. The claims and open-ended questions related to the future of teaching concentrated on the changes and consequences of pandemic time teachings. In total, the survey had 9 questions (multiple choice and open-ended questions) related to teachers’ background information and 24 Likert scale claims, 5 textboxes for justifying and specifying one’s choices for Likert scale claims, and 13 open-ended questions. For the details of these, please see the survey found in Online Appendix.

The survey was built on Microsoft Forms in Finnish and translated into English; both the versions were available for respondents. All the authors participated in commenting on content-related and linguistic issues in both Finnish and English, and both the versions were evaluated by a researcher with experience in survey studies. Due to the time constraints, pilot study could not be conducted. Thus, the final survey is constructed, evaluated, and/or commented by four experienced researchers who all have a PhD in the field of education. This cooperation of experts also enhances the validity and reliability of the instrument used.

### Distribution of the survey and sample

Finland has 13 universities and physics courses are taught in ten of them. The distribution of the survey took place in three forms. The main author gathered email addresses from university webpages according to their titles, so that university lecturers, university teachers, etc. were reached. For reaching professors, associate professors, and researchers that are teaching besides their research duties, the survey was sent for the heads of departments and the representatives of physics discipline with a request for distributing the survey furthermore. Besides, the Microsoft Teams group of a national network of people interested in developing teaching and learning of physics at university was used. The survey was sent for more than one hundred teachers via email, 15 heads of departments or representatives of physics discipline, and shared in Microsoft Teams for 34 members. It should be noted that these sharing methods were not exclusive in terms of recipients so the same people could have received it more than once. Besides, there is no information about how many heads of departments have forwarded the survey for their teaching staff, so we do not know that how many teachers have received the survey.

The survey was open for 1 month in September 2021 and 52 answers were received. 69% of the respondents answered the Finnish version of the survey and 31% the English version. As the survey distribution was partially outsourced for the departments, we cannot say what percentage of teachers reached responded the survey. However, we estimate it to be 30–40% as we assume to have reached the majority of teachers via direct emails.

Responses were received from nine universities. The distribution of responses from the different universities and the titles of respondents is omitted for the sake of respondents’ anonymity. All but three respondents had a doctoral degree, and teachers taught forthcoming physics and primary school teachers, researchers, engineers, and minor students. Distributions of the answers to the questions related to teaching experience presented in Table 2 show that in average teachers have had a rather long teaching experience. Teachers’ experiences related to the typical features of utilising online tools in teaching before the pandemic seen in Table 3 signal that teachers have had limited experience in utilising online tools in general.

**Table 2:** Respondents’ teaching experience at university level and other levels

Experience	At university level	Elsewhere
0–1 years	2	41
1–5 years	4	9
5–10 years	11	0
10–20 years	13	2
More than 20 years	22	0

*N* = 52

**Table 3:** Teachers’ experiences related to certain typical features of utilising online tools in teaching

	Not at all	0–1 years	1–3 years	3–5 years	More than 5 years
Sharing material for students via internet	1	3	4	7	37
Taking assignment returns in electronically	6	4	10	6	26
Synchronised teaching (e.g., lecturing) over internet	39	4	7	1	1
Self-made pre-recorded teaching videos	40	4	4	1	3
Supervising students over internet	26	4	7	6	9
Discussion platforms for students (email excluded)	29	7	8	2	6
Arranging and supervising of small group working over internet	36	6	4	2	4

*N* = 52

Teachers’ descriptions about their teaching during the pandemic revealed that they had utilised live lecturing and homework sessions via internet, pre-recorded videos, exams made at home or in specially equipped individual exam rooms on-campus, or substituting exams with essay tasks, and self-assessment. Small group working on-campus has been used in experimental working when allowed; in other cases, simulations, sending pre-measured data, and experiments done with home equipment have been used.

### Data analysis

Our data consists of teachers’ choices for the Likert scale claims and their answer to the open-ended questions. These data sets were analysed quantitatively and qualitatively, and hence this research includes data and method triangulation (Thurmond 2001).

Distributions concerning teachers’ choices for Likert scale claims are presented in relative proportions to the number of respondents. In certain claims, teachers’ choices “Totally disagree” and “Disagree” correspond to negative views and in certain claims to positives views; this feature is illustrated with the aid of different patterns in Results.

Teachers’ responses to open-ended questions were categorised by following the principles of theory-guided content analysis (Kohlbacher 2016). Researchers’ familiarity with the previous findings guided the process of finding repetitive themes from teachers’ responses. These themes were specified to formulate categories that all teachers’ responses were placed in. Each answer can be placed in more than one category. After the categorisation process conducted by Author 1, the categorisation was checked by either Author 2 or Author 3, and possible conflicts were discussed until a satisfactory consensus was reached. The consensus percentage after the first categorisation was 84%. A big portion of the categorisations that were questioned by Authors 2 and 3 were related to specifying category names so that the categorisation would be unambiguous, and categories would be exclusive. The final categorisation was satisfactory for all the authors. Hence, investigator triangulation was utilised to improve the trustworthiness of the study. Categories with less than three responses are not introduced for the sake of pithiness.

### Data availability statement

The data that support the findings of this study are not openly available due to securing the anonymity of the respondents and their institutions. However, anonymised data sets are available from the corresponding author upon reasonable request.

## Results

Results are presented thematically as follows: using technology, interaction, learning and evaluation, experimental working, other challenges and opportunities, and future (see Table 1). First, the distributions for Likert scale claims and categorisations of teachers’ responses to open-ended questions are introduced. Then, the most essential findings emerging from these are discussed.

### Using technology

**Fig. 1:**
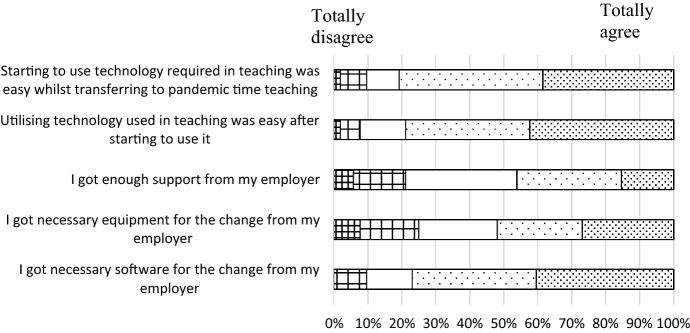
Teachers’ choices for Likert scale claims related to using technology. The scale goes from left to right as follows: totally disagree, disagree, neutral, agree, and totally agree. Dot patterns refer to positive choices, square patterns refer to negative choices, and white refers to neutral choices. *N* = 52

Figure 1 shows the distribution of teachers’ choices for five Likert scale claims and Table 4 shows the categorisation of their responses to the open-ended questions related to teachers’ views and experiences concerning the use of technology.

**Table 4:** Categorisation of teachers’ open-ended responses concerning the challenges and opportunities provided by technology

Challenges (*N* = 33)	Opportunities (*N* = 34)
Functionality and limitations of technology or internet, 14 responses “Sometimes there was connectivity issues with Wi-Fi dropping momentarily, but reconnection was smooth”	Flexibility towards time and space independent teaching, 13 responses “Recorded videos of lectures. Participating from other locations than the university city.”
The lack of students' participation and interaction in the remote teaching, 8 responses “Interaction with students is much more challenging”	Technology provided students better opportunities to follow and participate in teaching, 7 responses “iPad and a pen: same as a whiteboard in a lecture hall. Interactive tasks during lecture: makes one to process the issue at hand immediately, and one can see from the distribution that also numerous other students answer it wrong.”
Getting familiar with new technology, 5 responses “At first one had to study new things—it was not difficult but required time and effort.”	The use of technology felt convenient for teachers, 7 responses “It was nice to stream whiteboard lectures. It functioned well.”
Students' inabilities to use remove teaching technology due to lack of their skills or equipment, 4 responses “The most challenges considered the technological problems of the students (i.e., they did not use webcams, not even microphones, or they had problems with Internet connection, using Zoom, etc..)”	Distributing and returning assignments and other materials electronically, 6 responses “Distributing all material to both directions.”
No challenges, 3 responses “No specific problems come to my mind.”	Vague, 4 responses “Learned new tricks useful for future.”

Each category is followed by the number of responses in that category and the representative quotes

Figure 1 shows that teachers’ experiences about using technology and getting support for it are on a positive side. Teachers’ specifications revealed that there have been some practical issues with the availability and payment of equipment and the applications used by universities. The categorisation of teachers’ open-ended answers (Table 4) supplements these findings by illustrating that the greatest challenges have emerged from the functionality of technology and communications. Flexibility and detailed help provided by technology are highlighted as opportunities.

### Interaction

**Fig. 2:**
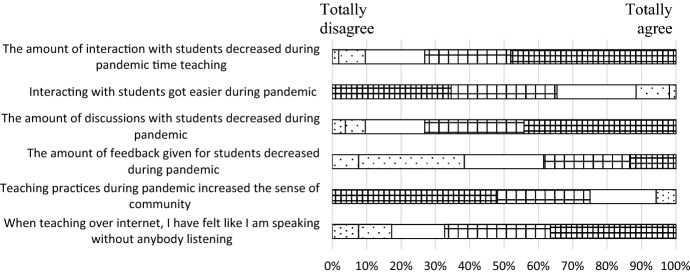
Teachers’ choices for the Likert scale claims related to interaction. The scale goes from left to right as follows: totally disagree, disagree, neutral, agree, and totally agree. Dot patterns refer to positive choices, square patterns refer to negative choices, and white refers to neutral choices. *N* = 52

The distribution of teachers’ responses for the five Likert scale claims concerning the changes taken place in interaction is seen in Fig. 2. The categorisation of their responses to open-ended questions for the related challenges and opportunities is seen in Table 5.

**Table 5:** Categorisation of teachers’ open-ended responses concerning the challenges and opportunities related to interaction

Challenges (*N* = 33)	Opportunities (*N* = 35)
Interaction became absent, less frequent, or more difficult, 16 responses “There is no interaction at all. Students hide behind black screens. There is no even indication that they watch/listen lectures.”	Opportunities given by technology, 11 responses “Including different medias for teaching became more flexible.”
The lack of seeing students or their reactions, 6 answers “We cannot read the feeling or reaction of the student. So, it is impossible to adapt the lecture level.”	Interaction worked well with small groups or individual students, 5 responses “One-on-one sessions for guiding students in their use of course-designated design software was an efficient way of helping the students out with technical issues in their exercises/projects.”
Problems caused by technology, 4 responses “Sometimes the sound quality has room for improvement.”	Only minor opportunities for interaction, 4 responses “Students were totally passive in Zoom lectures, especially when they were recorded from students’ request. Only chat brought some comments. In an advanced-level course that wasn’t recorded, students were at least a bit active.”
Students had to be persuaded, 4 responses “Students had to be (and still have to) persuaded to take contact in group teaching situations and lectures. Everything goes much better with two people.”	Saving time/flexibility, 5 responses “Remote teaching offers great possibilities for added flexibility, both for the teachers and for the students. This is an aspect I think can be developed further to improve the quality of teaching in general.”
	Students did not use the possibilities offered, 3 responses “Chat and email could have been used but they weren’t.”
	No any opportunities for interaction, 6 responses “No new better possibilities.”

Figure 2 shows that teachers have experienced negative changes in almost all the aspects of interaction during the pandemic. Teachers’ justifications and specifications for their choices revealed some contradictory views: some teachers expressed that the number of questions and comments increased in online teaching as the threshold using chat was smaller than saying things aloud, and other teachers stated that attempts to enhance interaction failed. Teachers’ open-ended responses seen in Table 5 strengthen these findings, and the decrease in the amount of interaction becomes evident, even if both the positive and negative aspects of technology for interaction are seen in open-ended responses.

### Learning and evaluation

**Fig. 3:**
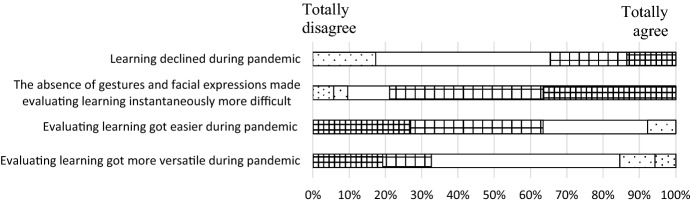
Teachers’ choices for Likert scale claims related to learning and evaluation. The scale goes from left to right as follows: totally disagree, disagree, neutral, agree, and totally agree. Dot patterns refer to positive choices, square patterns refer to negative choices, and white refers to neutral choices. *N* = 52

The distribution concerning teachers’ responses for the four claims related to learning and evaluation is introduced in Fig. 3, whilst the categorisation of their open-ended responses is seen in Table 6.

**Table 6:** Categorisation of teachers’ open-ended responses concerning the challenges and opportunities related to learning and evaluation

Challenges (*N* = 26)	Opportunities (*N* = 22)
Lack of control in exams and other assignments during remote teaching, 8 responses “There was cheating in email exams, mostly working with a pair. For sure, internet and lecture materials were also used.”	More versatile evaluation methods (e.g., the use of common exam spaces, oral exams, randomisation in electronic exams), 6 responses “We could more naturally promote continuous assessment with smaller electronic tests along the course.”
Problems emerging from the lack of interaction or observing students, 5 responses “The lack of personal contact.”	No need for changes, 5 responses “Remote teaching did not offer any possibilities that was not in use already before.”
Electronic evaluation with feedback increased workload, 4 responses “It takes much more time when there’s more of evaluating written assignments.”	Opportunities emerging from remote teaching, 3 responses “Exams made at home.”
Formulating suitable exam questions and assignments for remote teaching, 3 responses “We used partly open-book home exams, which required a new type of questions that cannot be simply googled.”	Problems emerging from the lack of interaction or observing students, 5 responses “The lack of personal contact.”
No challenges, 5 responses “Evaluating the students was exactly the same, since it is based on the exercises and exam problems they do, not on how they interact during the lectures.”	Nothing, 5 responses “None.”

Figure 3 shows that numerous teachers have expressed either uncertainty or mixed views concerning student learning and versatility of evaluation. The general picture concerning teachers’ views concerning learning and evaluation is rather negative. Teachers’ specifications and justifications for their choices addressed both new possibilities enabled by technology and concerns related to it. Teachers’ open-ended responses seen in Table 6 support these findings and bring some details: Problems related to making suitable tasks for remote teaching and exams and possibilities of cheating were mentioned repeatedly as challenges. New tools or methods for evaluation were highlighted as opportunities.

### Experimental working

**Fig. 4:**
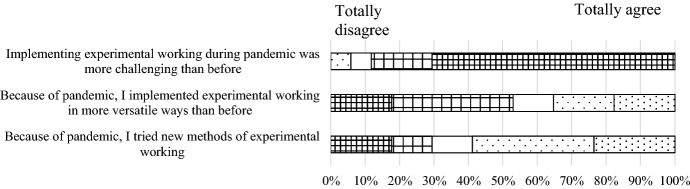
Teachers’ choices for the Likert scale claims related to experimental working. The scale goes from left to right as follows: totally disagree, disagree, neutral, agree, and totally agree. Dot patterns refer to positive choices, square patterns refer to negative choices, and white refers to neutral choices. *N* = 17

Figure 4 summarises teachers’ responses for the claims related to experimental working. These questions were directed for the teachers who have had experimental working, and therefore, the number of respondents is smaller than in other questions. The categorisation of teachers’ responses for open-ended questions is presented in Table 7.

**Table 7:** Categorisation of teachers’ open-ended responses concerning the challenges and opportunities related to experimental working

Challenges (*N* = 7)	Opportunities (*N* = 13)
It was forbidden, 3 responses “It was prohibited, thus only model data.”	Experimental working with home equipment and simulations, 3 responses “It was easier to sell an experimental work for students when it is made with one’s one equipment”
Inadequate experimental equipment found at students' home, 3 responses “Experimental research related to modern physics phenomena with home equipment.”	The use of video recorded experiments and demonstrations, 2 responses “Lecture demonstrations and laboratory work were recorded”
The use of home-made experiments or reducing the laboratory work for the analysis of already measured data, 2 responses “A part of the lab assignments had to be changed to a remote mode.”	Increased states of freedom in assignments related to experimental working, 1 response “The freedom in many basic lab experiments increased (regarding both planning and measurements), thus I think that some of the lab exercises are even better in their current form (e.g., considering exercises connected to basic courses of physics, e.g., studying trajectory motion and air resistance).”
The expressions of difficultness and anxiety regarding the teaching of experimental work during the pandemic, 2 responses “It was pretty challenging.”	No opportunities, 5 responses ”I don’t think it gave any opportunities, mainly a poor substitute.”
Student dropout, 1 response “Students disappeared from the course.”	

Due to the small number of respondents, all categorized responses are presented

The distribution of the responses to the first Likert claim shows that experimental working during the pandemic has been more challenging. The following two claims with more positive responses can be considered as consequences of the first one; teachers reacted to the situation. Teachers’ specifications for their claims revealed that some experimental working was run as usual, just with smaller groups, and that some demonstrations worked better over the internet than others. Teachers’ open-ended responses categorised in Table 7 give details for the challenges and highlight some opportunities; interestingly, experimental working at home is addressed in both the categories.

### Other challenges and opportunities

A categorisation of teachers’ responses to the questions “What and what kinds of other challenges have pandemic time teaching brought?” and “What and what kinds of opportunities have pandemic time teaching brought?” is seen in Table 8.

**Table 8:** Categorisation of teachers’ open-ended responses concerning other challenges and opportunities

Challenges (*N* = 25)	Opportunities (*N* = 24)
Teachers’ well-being, 7 responses “Sometimes it is difficult to cope whilst working days are long, and there is no distinction between work and free time.”	Using and testing different ways of remote teaching, 7 responses “We have seen and tested a larger range of electronic tools to help teaching—and improved our electronic course homepages.”
Feedback and different modes of interaction, 7 responses “Informal interaction between teachers has decreased, and hence the ideas have not spread.”	Better accessibility for materials, 5 responses “Possibly reaching bigger student groups.”
Working at home, 5 responses “Teaching at home when there are primary level pupils studying at home. Instability of internet connection. Background noise from neighbours or outside. Problems with equipment (microphone etc.).”	Supplementing other instruction, 3 responses “New functional things should be combined with traditionally functional ones, i.e., taking good sides from both for implementing courses. Pandemic time has made it concrete that numerous things can still be developed.”
Students’ well-being and coping with studies, 4 responses “A bigger portion of students than normally feel bad.”	Technology-related opportunities, 3 responses “I guess some technological things can be utilized later”
More effort was needed, 3 responses “Timing issues with instruction and other work duties”	No opportunities, 4 responses “I cannot think of any at the moment.”

Each category is followed by the number of responses in that category and representative quotes when possible

The most interesting finding, besides bringing some details for the themes introduced earlier, is highlighting the problems in well-being of teachers and students as the pandemic time has evidently been harsh for both the teachers and students. Some details of the opportunities, such as the novel use of technology and adding diversity, were emphasised in the open-ended responses.

### Future

**Fig. 5:**
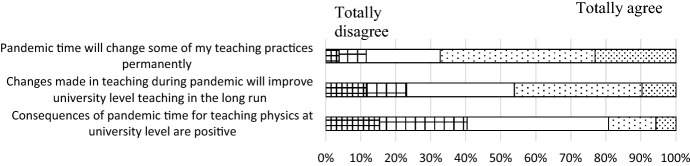
Teachers’ choices for Likert scale claims related to the future of teaching in post-pandemic time. The scale goes from left to right as follows: totally disagree, disagree, neutral, agree, and totally agree. Dot patterns refer to positive choices, square patterns refer to negative choices, and white refers to neutral choices. *N* = 52

The distribution of teachers’ choices for three future-related Likert scale claims is seen in Fig. 5. A categorisation of teachers’ responses to the questions “What sorts of changes in your teaching will remain as a consequence of pandemic?” is shown in Table 9.

**Table 9:** Categorisation of teachers’ open-ended responses concerning the changes in their instruction as a consequence of the pandemic

Changes for instruction (*N* = 24)
Continuing remote teaching to some degree, 8 responses “Part of teaching might remain in remote mode also in future, and in contact teaching the emphasis will be more on utilising time valuably for interaction.”
Distributing material, 6 responses “I might continue distributing materials in Teams even if I had contact teaching.”
Utilising videos, 5 responses “I will add different sorts of videos related to the topics of my courses in my course webpage.”
Online possibilities in supervision, 3 responses “I dare to use video calls more often, for example in supervising theses.”
Versatility for instruction, 3 responses “More versatile teaching and supervising.”

More than a half of the teachers stated to change their teaching practices, which signals teachers to have found functional teaching practices. Besides, improvements for university level teaching in the long run were also recognized. Despite these, consequences to teaching physics at university level are seen negatively. Teachers’ specifications for their choices showed that teachers saw it positively that teachers had to change their practices with positive outcomes. Generally, teachers seemed to have mixed feelings and experiences about pandemic time teaching. The following important findings are found in open-ended responses; utilising remote teaching and videos to make teaching more versatile and to provide supplementary material.

### Answering the research questions

In order to summarize our results, answers to the research questions are presented. The first research question was stated “What sorts of challenges did university teachers face in teaching physics during the pandemic?”. Generally, technology did not seem to challenge teachers even if some concerns were highlighted. Interaction was seen as a major issue that posed significant challenges for teachers. Teachers’ responses related to the learning and evaluation showed that even if the decline in learning was not seen as a major issue, challenges were posed by home-made exams, not seeing students’ gestures, and risks of cheating. The challenges related to experimental working indicate that because laboratory working has been mostly forbidden during the pandemic, the absence has been the major challenge. Other challenges reported by teachers are related to the well-being of themselves and students under special circumstances.

The second research question was formulated “What sorts of possibilities did university teachers experience in teaching physics during the pandemic?”. Flexibility, enhancing teaching, and electronic tools were the most prominent technology-related opportunities highlighted. Regarding interaction, using different media and working in small groups were seen somewhat positively even if the general view was negative. Teachers did not highlight many possibilities concerning learning and evaluation; there were only few mentions about new evaluation tools. Experimental working was not seen to offer much of possibilities. Nevertheless, some teachers mentioned that they had found ways to implement it under special circumstances. Besides, teachers mentioned both the accessibility and versatility of instruction as possibilities.

The third research question was formulated as “What sorts of changes will remain in teaching physics at university due to the pandemic?”. Our findings signal that a majority of teachers will change their teaching practices, even if the consequences of pandemic time for university level instruction are not seen that positively. The most common changes are related to continuing remote teaching and instruction at some situations, sharing material, and adding versatility of instruction.

## Discussion

In this article, university physics teachers’ experiences concerning teaching during the pandemic were evaluated with the aid of an online survey. The focus was on the challenges and possibilities experienced and possible changes for instruction remaining after the pandemic.

Related to the first research question, our finding about teachers not experiencing challenges with using technology is in agreement with the study from Lemay et al. (2020) but findings related to lacking technological skills are not supported (O’Brien 2021; Trust and Whalen 2021). The challenges in interaction is supported by previous research (Dew et al. 2021; Romadhon et al. 2021; Trust and Whalen 2021). Partially conflicting findings related to interaction from Kannan et al. (2020) and Lemay et al. (2020) might be explained with different ways of instruction and data gathering methods. In this research, teachers’ views concerning learning and teaching seem to be rather negative in comparison to previous research, even if some similar concerns are introduced by Lemay et al. (2020). Because experimental working has been either forbidden or limited during the pandemic, it has been expectedly stated to be a great challenge also in previous research (O’Brien 2021; Romadhon et al. 2021; Nuere and de Miguel 2020). We should point it out that Ametepe and Khan (2021) and Jelicic et al. (2022) have given research-based recommendations for functional online and hybrid laboratory courses, which gives a more positive outlook for the issue. Our finding concerning well-being of the teachers is supported by Klapproth et al. (2020) who have reported that teachers from various school levels have experienced medium to high levels of stress during the pandemic.

Regarding the possibilities addressed in the second research question, teachers emphasised flexibility, enhancing teaching, and tools similarly than reported by Lemay et al. (2020) and Tsegay et al. (2022). Possibilities related to interaction, namely utilising medias and working with small groups, is supported by the previous research (Dew et al. 2021; Romadhon et al. 2021). However, the general picture of interaction during the pandemic is more negative in our study than in some other previous studies (Lemay et al. 2020). Our finding suggesting that the pandemic time offered only minor possibilities for learning and evaluation is supported by Lemay et al. (2020) and Rapanta et al. (2020).

The third research question related to types of changes remaining in one’s instruction due to the pandemic seem to address a topic that has not received much of attention previously. However, one of our essential findings, namely increasing the versatility of physics instruction via remote teaching technologies, is also recommended by Australian Institute of Physics (Schröder-Turk and Kane 2020). This is an interesting finding that suggests that teachers have lacked either time, resources, or information of modern learning environments, even if they value them later.

The choice of using both quantitative and qualitative data enhances the trustworthiness of the study as the same research questions are approached with two data sets and analysis methods (Creswell 2009; Thurmond 2001).

The online survey was constructed by three experienced researchers (authors) who have PhDs in physics education research, and the final version was checked and commented by an evaluator with a doctoral degree from the field of education. The construction process with literature references is described carefully in Methods and the full version of the survey is found in Online Appendix so a reader can evaluate the process and the survey.

Analysing the Likert scale data were straightforward and free of interpretations. The addition of patterns to illustrate positive and negative choices is made discretionally based on the literature review and authors’ expertise, and the role of that is to ease seeing the main findings without losing details.

Teachers’ answers to the open-ended questions were more open for interpretations. The trustworthiness of the analysis was enhanced by means of investigator triangulation when the categorisation was checked by another researcher, and possible conflicts were discussed. The categorisation itself was a theory-guided content analysis (Kohlbacher 2016). Authentic quotes are presents in Results to give a better view about the nature of categories which aims at giving a rich, thick description of the topic. (Creswell 2009).

University physics instruction is likely to become more versatile after the COVID-19 pandemic since the teachers have familiarized themselves with remote teaching technology, even if some teachers might not have grasped the new methods and tools for successful online instruction. Since the versatility increases the possible modes of instruction, teachers still need deeper understanding and training of what is required for effective physics instruction so that they can better match appropriate instructional modes for the learning aims. Discipline-based educational research seems to be a recommendable starting point for this, since it covers various aspects of teaching and learning of physics (Docktor and Mestre 2014). Because physics instruction depends on local conditions, such as teaching facilities, teachers’ personal preferences, and students’ pre-knowledge, we recommend that university teachers should have freedom to decide instructional modes to be used instead of major university level guidelines. We agree with the Australian Institute of Physics (AIP) that online teaching should not totally replace face-to-face and hands-on curricula (Schröder-Turk and Kane 2020). Online delivery can be cost-effective and be used to reach bigger cohorts, but drop-off rates might get higher (Delgado 2021) and the quality of learning is still a major open question (Hasan and Odja 2021; Kannan et al. 2020). Therefore, teachers’ voice and ownership should have a high priority in designing university physics instruction. Besides, the well-being of teachers should also be remembered as it gets easily compromised in changing process.

It would be interesting to make similar studies in other science disciplines and lower school levels. We think that our survey gives a good starting point to make discipline-specific surveys that would take their special distinct features into account. We also think that studying actual changes in teachers’ practices some years later might give an important insight related to the consequences of the pandemic time. Besides, well-being of teachers and students is important and interesting research topic as the consequences of the pandemic time might be long-lasting.

## Supplementary Information

Below is the link to the electronic supplementary material.

Supplementary file1 (DOCX 24 KB)

## Data Availability

The data that support the findings of this study are not openly available due to securing the anonymity of the respondents and their institutions. However, anonymized data sets are available from the corresponding author upon reasonable request.
