# Supplementary material for: Finnish university physics teachers’ experiences of transferring to online teaching due to COVID-19 pandemic
Source: SN Soc Sci. 2023 Mar 23;3(4):68. doi: 10.1007/s43545-023-00647-1 (PMC10034880; doi:10.1007/s43545-023-00647-1)
Supplement: Supplementary file 1 — Supplementary file1 (DOCX 24 KB) [file 43545_2023_647_MOESM1_ESM.docx]

Appendix. The survey used in the study.

**A questionnaire related to physics teaching during pandemic (COVID-19)**

This questionnaire is used to gather research data about university level physics teachers' experiences concerning teaching during pandemic. The questionnaire is implemented by the physics and mathematics education research group in University of Eastern Finland.

Answering the questionnaire is voluntary, and the data is handled confidentially and stored securely. At the end, voluntary participants for research interviews are asked with the aid of email addresses. Only the responsible researcher (Risto Leinonen) has access to these email addresses. Before any reporting, all the data will be anonymised. According to GDPR, a respondent has a right to see the information stored related to him/her whilst asked.

Answering the questionnaire takes approximately 15 minutes.

Thank you for your responses!

More information:
*AUTHOR INFORMATION*

*Background information*

In this section, necessary background information of participants is addressed.

1. Your highest degree?

- Bachelor of science
- Master of science
- Licentiate of philosophy
- Doctor of philosophy
- Graduate engineer (diplomi-insinööri)

1. In which university you are working in? If you work in more than one university, choose all the ones where you are teaching at.

- Aalto University
- University of Helsinki
- University of Eastern Finland
- University of Jyväskylä
- University of Lapland
- Lappeenranta–Lahti University of Technology LUT
- University of Oulu
- Hanken School of Economics
- University of the Arts Helsinki
- Tampere University
- University of Turku
- University of Vaasa
- Åbo Akademi University

1. What is your title? You can choose more than one option. If you cannot find your exact title, choose the most appropriate one or write your title to the field Other.

- Part-time teacher
- University teacher
- Lecturer
- University lecturer
- Senior university lecturer
- Early stage researcher
- Postdoctoral researcher
- Senior researcher
- Associate professor
- Professor
- Project researcher
- Research director
- Research manager
- Research assistant
- Docent

1. How long is your teaching experience at the university level?

- 0-1 years
- 1-5 years
- 5-10 years
- 10-20 years
- More than 20 years

1. How long is your teaching experience elsewhere than in university?

- 0-1 years
- 1-5 years
- 5-10 years
- 10-20 years
- More than 20 years

1. To whom are you teaching physics at university level? You can choose more than one option.

- Forthcoming researchers
- Forthcoming physics teachers
- Forthcoming class teachers
- Forthcoming graduate engineers (diplomi-insinööri)
- Other, what?

1. How much experience did you have about the following before pandemic (March 2020)?

|  | Not at all | 0-1 years | 1-3 years | 3-5 years | More than 5 years |
| --- | --- | --- | --- | --- | --- |
| Sharing material for students via internet |  |  |  |  |  |
| Taking assignment returns in electronically |  |  |  |  |  |
| Synchronised teaching (e.g. lecturing) over internet |  |  |  |  |  |
| Self-made pre-recorded teaching videos |  |  |  |  |  |
| Supervising students over internet |  |  |  |  |  |
| Discussion platforms for students (email excluded) |  |  |  |  |  |
| Arranging and supervising of small group working over internet |  |  |  |  |  |

1. You can supplement your previous answers in this field.
2. Tell shortly, how you have implemented your teaching during pandemic.

*Using technology*

In this section the challenges and possibilities posed by technology related to pandemic time teaching are focused on.

1. Evaluate your teaching and the related changes taken place during pandemic. Choose the most appropriate option based on your view.

|  | 1 = Totally disagree | 2 = Disagree | 3 = Neutral | 4 = Agree | 5 = Totally agree |
| --- | --- | --- | --- | --- | --- |
| Starting to use technology required in teaching was easy whilst transferring to pandemic time teaching |  |  |  |  |  |
| Utilising technology used in teaching was easy after starting to use it |  |  |  |  |  |
| I got enough support from my employer |  |  |  |  |  |
| I got necessary equipment for the change from my employer |  |  |  |  |  |
| I got necessary software for the change from my employer |  |  |  |  |  |

1. You may justify or specify your choices for the previous claims.
2. What sorts of challenges did you face with technology in remote teaching?
3. What sorts of opportunities did technology offer for remote teaching?

*Interaction*

In this section, different aspects of interaction during pandemic are addressed.

1. Answer the following claims by choosing the most appropriate option based on your view.

|  | 1 = Totally disagree | 2 = Disagree | 3 = Neutral | 4 = Agree | 5 = Totally agree |
| --- | --- | --- | --- | --- | --- |
| The amount of interaction with students decreased during pandemic time teaching |  |  |  |  |  |
| Interacting with students got easier during pandemic |  |  |  |  |  |
| The amount of discussions with students decreased during pandemic |  |  |  |  |  |
| The amount of feedback given for students decreased during pandemic |  |  |  |  |  |
| Teaching practices during pandemic increased the sense of community |  |  |  |  |  |
| When teaching over internet, I have felt like I am speaking without anybody listening |  |  |  |  |  |

1. You may justify or specify your choices for the previous claims.
2. What sorts of challenges did you face in the interaction with students in remote teaching?
3. What sorts of opportunities did remote teaching offer for interacting with students?

*Learning and evaluation*

In this section, the focus is on learning and evaluating it during pandemic time.

1. Answer the following claims by choosing the most appropriate option based on your view.

|  | 1 = Totally disagree | 2 = Disagree | 3 = Neutral | 4 = Agree | 5 = Totally agree |
| --- | --- | --- | --- | --- | --- |
| Learning declined during pandemic |  |  |  |  |  |
| The absence of gestures and facial expressions made evaluating learning instantaneously more difficult |  |  |  |  |  |
| Evaluating learning got easier during pandemic |  |  |  |  |  |
| Evaluating learning got more versatile during pandemic |  |  |  |  |  |

1. You may justify or specify your choices for the previous claims.
2. What sorts of challenges did you face in evaluations in remote teaching?
3. What sorts of opportunities did remote teaching offer for evaluation?
4. In which ways did you implement course evaluations during pandemic? How did this differ from your previous evaluations?

*Experimental working*

In this section the focus is on challenges and opportunities of experimental working that typically requires contact teaching.

1. Have you taught experimental working (e.g. supervised laboratory work) during pandemic? If you answer "No", move to the next section (question 28)

- Yes
- No

1. Answer the following claims by choosing the most appropriate option based on your view.

|  | 1 = Totally disagree | 2 = Disagree | 3 = Neutral | 4 = Agree | 5 = Totally agree |
| --- | --- | --- | --- | --- | --- |
| Implementing experimental working during pandemic was more challenging than before |  |  |  |  |  |
| Because of pandemic, I implemented experimental working in more versatile ways than before |  |  |  |  |  |
| Because of pandemic, I tried new methods of experimental working |  |  |  |  |  |
| Using simulations increased during pandemic |  |  |  |  |  |
| Experimental working done with household equipment increased during pandemic |  |  |  |  |  |
| I sent pre-measured data for students to be analysed and reported during pandemic |  |  |  |  |  |

1. You may justify or specify your choices for the previous claims.
2. What sorts of challenges did you face in experimental working during pandemic?
3. What sorts of opportunities did remote working offer for experimental working?

*Other*

In the questionnaire, challenges and possibilities related to using technology, interaction, learning and evaluation, and experimental working during pandemic have been addressed. In this section, your experiences not fitting under those themes are addressed.

1. What and what kinds of other challenges have pandemic time teaching brought?
2. What and what kinds of opportunities have pandemic time teaching brought?

*Future*

1. Answer the following claims by choosing the most appropriate option based on your view.

|  | 1 = Totally disagree | 2 = Disagree | 3 = Neutral | 4 = Agree | 5 = Totally agree |
| --- | --- | --- | --- | --- | --- |
| Pandemic time will change some of my teaching practices permanently |  |  |  |  |  |
| Changes made in teaching during pandemic will improve university level teaching in the long run |  |  |  |  |  |
| Consequences of pandemic time for teaching physics at university level are positive |  |  |  |  |  |

1. You may justify or specify your choices for the previous claims.
2. What kinds of changes for your teaching will remain as a consequence of pandemic time?
3. What else would you wish to say?
